# Modulation of Cellular Mg^2+^ Content in Cardiac Cells by α_1_-Adrenoceptor Stimulation and Anti-Arrhythmic Agents

**DOI:** 10.2174/1872208311206030212

**Published:** 2012-12

**Authors:** Andrea M.P Romani

**Affiliations:** Dept. Physiology and Biophysics, Case Western Reserve University

**Keywords:** Arrhythmias, Mg^2+^ extrusion, heart, cardiac ventricular myocytes, adrenergic agonist, catecholamine, quinidine, patents.

## Abstract

Magnesium (Mg^2+^) is used pharmacologically to sedate specific forms of arrhythmias. Administration of pharmacological doses of catecholamine or adrenergic receptor agonists often results in arrhythmias onset. Results from the present study indicate that stimulation of cardiac adrenergic receptors elicits an extrusion of cellular Mg^2+^ into the extracellular space. This effect occurs in both perfused hearts and isolated cells within 5-6 min following either β- or α_1_-adrenergic receptor stimulation, and is prevented by specific adrenergic receptors antagonists. Sequential stimulation of the two classes of adrenergic receptor results in a larger mobilization of cellular Mg^2+^ provided that the two agonists are administered together or within 1-2 min from each other. A longer delay in administering the second stimulus results in the abolishment of Mg^2+^ extrusion. Hence, these data suggest that the stimulation of β- and α_1_-adrenergic receptors mobilizes Mg^2+^ from two distinct cellular pools, and that Mg^2+^ loss from either pool triggers a Mg^2+^ redistribution within the cardiac myocyte. At the sarcolemmal level, Mg^2+^ extrusion occurs through a Na^+^/Mg^2+^ exchange mechanism phosphorylated by cAMP. Administration of quinidine, a patent anti-arrhythmic agent, blocks Na^+^ transport in a non-specific manner and prevents Mg^2+^ extrusion. Taken together, these data indicate that catecholamine administration induces dynamic changes in total and compartmentalized Mg^2+^ pools within the cardiac myocytes, and suggest that prevention of Mg^2+^ extrusion and redistribution may be an integral component of the effectiveness of quinidine and possibly other cardiac anti-arrhythmic agents. Confirmation of this possibility by future experimental and clinical studies might result in new patents of these compounds as Mg^2+^ preserving agents.

## INTRODUCTION

Cardiac arrhythmias constitute a major etiological and epidemiological issue, which translates into 180,000 to 250,000 sudden cardiac deaths per year in the US only [1]. Clinical evidence supports a protective role of Mg^2+^ in cardiovascular diseases [2] including a specific sedating effect in particular forms of arrhythmias such a “torsades de pointes” [3]. Yet, the mechanism behind this protective effect is poorly understood. 

Magnesium is the second most abundant cation within mammalian cells, with a total concentration ranging between 16 to 20mM [4, 5]. A variety of experimental approaches and methodologies indicate that magnesium ions (Mg^2+^) are highly and equivalently concentrated within mitochondria, endoplasmic (sarcoplasmic) reticulum, and nucleus [4-6]. Cytoplasm represents a smaller but equally important pool of cellular Mg^2+^. In the cytoplasm, Mg^2+^ is predominantly bound to adenosine trisphosphate, phosphonucleotides and phospho-metabolites [7]. Because of the high cellular concentration, Mg^2+^ was thought to remain largely unmodified under various hormonal or metabolic conditions in keeping with its essential role of coenzyme for various metabolic functions [4,5]. This notion was overturned when different laboratories evidenced that a sizable amount of cellular Mg^2+^ is extruded from various cell types [see ref. 4 for a list] following hormonal or metabolic stimuli.

Extrusion of cellular Mg^2+^ via adrenergic stimulation is the most investigated process as it has been observed in a variety of cells [4] including cardiac myocytes [8-1]. The mechanism ultimately responsible for Mg^2+^ extrusion across the sarcolemma shows all the characteristics of an Na^+^-dependent exchanger that become activated upon phosphorylation by cAMP [4,8,10]. While the structure of this Na^+^/Mg^2+^ exchanger is not completely defined, its presence and operation have been confirmed in cardiac sarcolemmal vesicles [12,13]. Notably, the extrusion of Mg^2+^ through the putative Na^+^/Mg^2+^ exchanger is inhibited by few non specific agents including quinidine [14] , a class I anti-arrhythmic drug [15]. This observation indirectly supports the notion that blocking Mg^2+^ extrusion may represent an intrinsic component of the mechanisms of action for at least some of these therapeutic agents.

An ancillary issue is the identification of the compartment(s) from which Mg^2+^ is mobilized upon adrenergic stimulation. The presence of distinct but equivalent compartments within the cells raises the question as to whether different stimuli mobilize Mg^2+^ from different compartments, and whether cellular redistribution occurs as a result of specific adrenergic stimulation. Experimental evidence indicates that -adrenoceptor agonist resultαrepeated administrations of submaximal doses of in progressively smaller Mg^2+ ^extrusions from cardiac cells [8], hinting to the progressive depletion of a cellular pool. Experimental observation also suggests that the mitochondrial pool may represent one of the pools – if not the pool – from which β-adrenergic stimulation mobilizes Mg^2+^ [16,17]. On the other hand, administration of epinephrine to liver cells results in a Mg^2+^ mobilization that is quantitatively equivalent to the sum of the amounts mobilized by the distinct administration of α_1_- and β-adrenergic stimuli [18], further supporting the notion that distinct adrenergic stimuli mobilize Mg^2+^ from two quantitatively different cellular pools.

The lack of appropriate methodologies prevents from properly and dynamically determining how is Mg^2+^ mobilized from an intracellular compartment and whether cellular Mg^2+^ redistribution occurs during or following adrenergic stimuli. The present study was undertaken with the two fold intent of elucidating whether Mg^2+^ redistribution occurs following adrenergic stimulation and to which extent quinidine administration affects such a process. To address this question, we used hearts perfused in a Langendorf retrograde manner and stimulated by sequentially administering selective α_1_- and β-adrenergic agonists with a varying interval between stimuli application. The results reported in the present study suggest, albeit indirectly, that Mg^2+^ is rapidly redistributed within cellular compartment based upon the imposed stimuli sequence. Moreover, data reported in the present study indicate that commonly used anti-arrhythmic drugs block adrenergic-induced Mg^2+^ extrusion from cardiac cells. The latter observation is interpreted as a possible mechanism whereby preservation of cellular Mg^2+^ extrusion can exert auxiliary anti-arrhythmic effects in cardiac cells undergoing persistent adrenergic stimulation. 

## MATERIALS AND METHODS

Collagenase type I was from Worthington (Lakewood, NJ). Isoproterenol, phenylephrine, 8-Br-cAMP, and all chemicals used in the study were of analytical grade (Sigma, St Louis, MO). Male Sprague-Dawley rats (220-250 g body weight) were used as organ donors. The animals were maintained on 12 h light/no light cycle and had free access to food and water until used.

On the day of the experiment, the animals were anesthetized by i.p. injection of a saturated sodium pentobarbital solution (65 mg/kg b.w.). Following disappearance of corneal and pain reflexes the chest was opened and the heart rapidly excised at the aorta arch. The aorta was mounted on a truncated 16 gauge needle and the heart was flushed with a medium containing (mM): NaCl 120, KCl 3, CaCl_2_ 1, MgCl_2_ 0.8, K_2_HPO_4_ 1.2, NaHCO_3_ 12, glucose 15, Hepes 10, pH 7.2 at 37^o^C, equilibrated with an O_2_:CO_2_ (95:5, v/v) gas mixture (Perfusion medium) as previously detailed [8]. The heart was then connected to a perfusion system and retrograde perfused in a Langerdoff manner with the medium indicated above equilibrated with O_2_:CO_2_ (95:5, v/v) at a flow rate of 7 ml/g/min, at 37^o^C [8]. After a few minutes of equilibration, the perfusion medium was switched to one having a similar composition but devoid of Mg^2+^ (Mg^2+^-free medium). The contaminant Mg^2+^ present in the medium was measured by atomic absorbance spectrophotometry (AAS) in a Perkin-Elmer 3100, and found to range between 5 and 7 μM. Aliquots of the perfused medium were collected at 30 s intervals, and assessed for Mg^2+^ content by AAS. The first 10 min of perfusion provided a baseline for the subsequent addition of adrenergic agonist. Isoproterenol (10 μM), phenylephrine (5 μM) epinephrine (5 μM) or 8-Br-cAMP (250 μM) were directly dissolved into the perfusion medium, and administered for the time reported in the figures either individually or sequentially at different time intervals. Pharmacological doses of the adrenergic agonists were used throughout the study to exclude reduced adrenergic receptor responsiveness. To estimate the total amount of Mg^2+^ extruded from the organ, the Mg^2+^ content in the perfusate at the last six points prior to the adrenergic agonist administration was averaged and subtracted from each of the time points under the curve of efflux. The net amount of Mg^2+^ mobilized into the perfusate (nmol/ml) was then calculated taking into account the perfusion rate (7 ml/g /min) and the time of collection (30 s), and expressed as μmol [8]. 

In experiments in which amiloride (350μM), nifdipine (1μM), or prazosin (1μM) were used as non-specific inhibitors of the Na^+^/Mg^2+^ exchange mechanism, L-type Ca^2+^ channels, and α_1_-adrenoceptors, respectively, these agents were dissolved directly into the perfusion medium and administered for 5 min prior to the addition of the agonist, and maintained in the system throughout the agonist administration. 

Under all experimental conditions, the absence of cell damage was assessed by measuring LDH activity, Aliquots of the perfusate were collected at 1 min interval throughout the experimental procedure, and LDH activity measured by enzymatic kit (Sigma, St Louis, MO) sensitive to detect changes in the mU/ml range, and expressed as U/L. The release of K^+^ from potentially damaged cells was also measured by AAS in aliquots of the perfusate according to published protocol [8]. 

At the end of the perfusion procedure, the residual Mg^2+^ content within the perfused heart was also calculated in tissue homogenate. To this end, the heart was removed from the perfusion system, gently blotted on absorbing paper, and weighted to normalize Mg^2+^ extrusion per gram of tissue. The heart was homogenized (10% w/v) in 250mM sucrose using three cycles of 10 sec each in a Polytron at setting 3. The homogenate was then acidified by addition of 10% HNO_3_ (final concentration) [8]. 

### Determination of Cardiac Ion Content

After overnight digestion, aliquots of the acid homogenate extract were transferred in microfuge tubes, and the denaturated protein was sedimented at 8,000g for 5 min. The acid supernatants were removed and assessed for Mg^2+^, Ca^2+^, Na^+^, and K^+^ content by AAS after proper dilution. Cation content was normalized for protein content, measured according to the procedure of Lowry *et al.* [19], and for tissue weight.

### Cardiac Myocytes Isolation

Cardiac ventricular myocytes were isolated by collagenase digestion according to our published protocol [8]. After isolation, myocytes were resuspended in the perfusion medium indicated previously, at the final concentration of 2x10^5^ cells/ml containing 0.8 mM MgCl_2_, and kept at room temperature, under constant flow of O_2_:CO_2_ (95:5, v/v) until used. Cell viability, assessed by LDH release and maintenance of cardiac myocytes rod shape, was found to be 78±5% (n=9), and did not change significantly over the course of 4 h (77±7%, n=9). To determine Mg^2+^ transport, 1 ml of myocytes suspension was transferred to a microfuge tube, and the cells were rapidly sedimented at 700g x 30 s. The pellet was washed once with 1 ml of Mg^2+^-free medium. After the wash, the myocytes were transferred to 8 ml of Mg^2+^-free incubation medium, pre-warmed at 37^o^C, and incubated therein under continuous O_2_:CO_2_ flow and stirring. After 2 min of equilibration, isoproterenol, phenylephrine, epinephrine, or cell permeant cAMP were added to the incubation system at the concentrations indicated above. Following the agonist addition, 0.7 ml of incubation mixture was withdrawn in duplicate at 2 min intervals, and the cells sedimented in microfuge tubes. The supernatants were removed and assessed for Mg^2+^ content by AAS. The cell pellets were digested overnight in 10% HNO_3_. The denaturated protein content was sedimented at 8,000g x 5 min in microfuge tubes and the Mg^2+^ content of the acid extract was measured by AAS.

In experiments in which amiloride (300 μM), quinidine (250 μM), nifdipine (1 μM), or prazosin (1 μM) were used as non-specific inhibitors of the Na^+^/Mg^2+^ exchange mechanism, L-type Ca^2+^ channels, and α_1_-adrenoceptors, respectively, these agents were added to the incubation medium together with the cells, approximately 5 min prior to the addition of the adrenergic agonist. 

### Cellular Mg^2+^ Distribution

Estimation of total cellular Mg^2+^ content and distribution among cytoplasm, mitochondria, and other cellular organelles (mainly but not only sarcoplasmic reticulum) were carried out in cardiac myocytes incubated in Mg^2+^-free medium as described above. Digitonin (50 μg/ml final concentration), carbonyl cyanide p-trifluoromethoxyphenyl-hydrazone (FCCP, 2 μg/ml), and A23187 (2 μg/ml) were sequentially added to the incubation system at 5 min interval, and aliquots of the incubation mixture were withdrawn and sedimented in microfuge tubes at 10,000g x 2 min. The Mg^2+^ content of the supernatant was measured by AAS. Residual Mg^2+^ content in the cell pellets was measured by AAS after acid digestion performed as reported in the previous paragraph. The Mg^2+^ content present in the cell pellet or the extracellular space prior to the addition of any stimulatory agent was calculated and subtracted from the following time points of incubation to determine the net amount of Mg^2+^ retained within the cell or released into the incubation medium. Release of LDH from cardiac myocytes was measured enzymatically (kit from Sigma), and expressed as a percentage of the total amount of the enzyme releasable from digitonin-permeabilized cells. Cellular ATP content level was measured in isolated myocytes by luciferin-luciferase assay as reported previously [20]. 

### Animals Ethics

Animals were maintained and handled in accordance with the Guide for the Care and Use of Laboratory Animals (Institute of Laboratory Animal Resources, Commission on Life Science, National Research Council 1996), as approved by the Animal Resource Center at Case Western Reserve University, Cleveland, Ohio.

### Statistical Analysis

The data are reported as mean±SE. Data were first analyzed by one-way ANOVA. Multiple means were then compared by Tukey’s multiple comparison test performed with a q value established for statistical significance of at least P<0.05.

## RESULTS

**Fig. (1):**
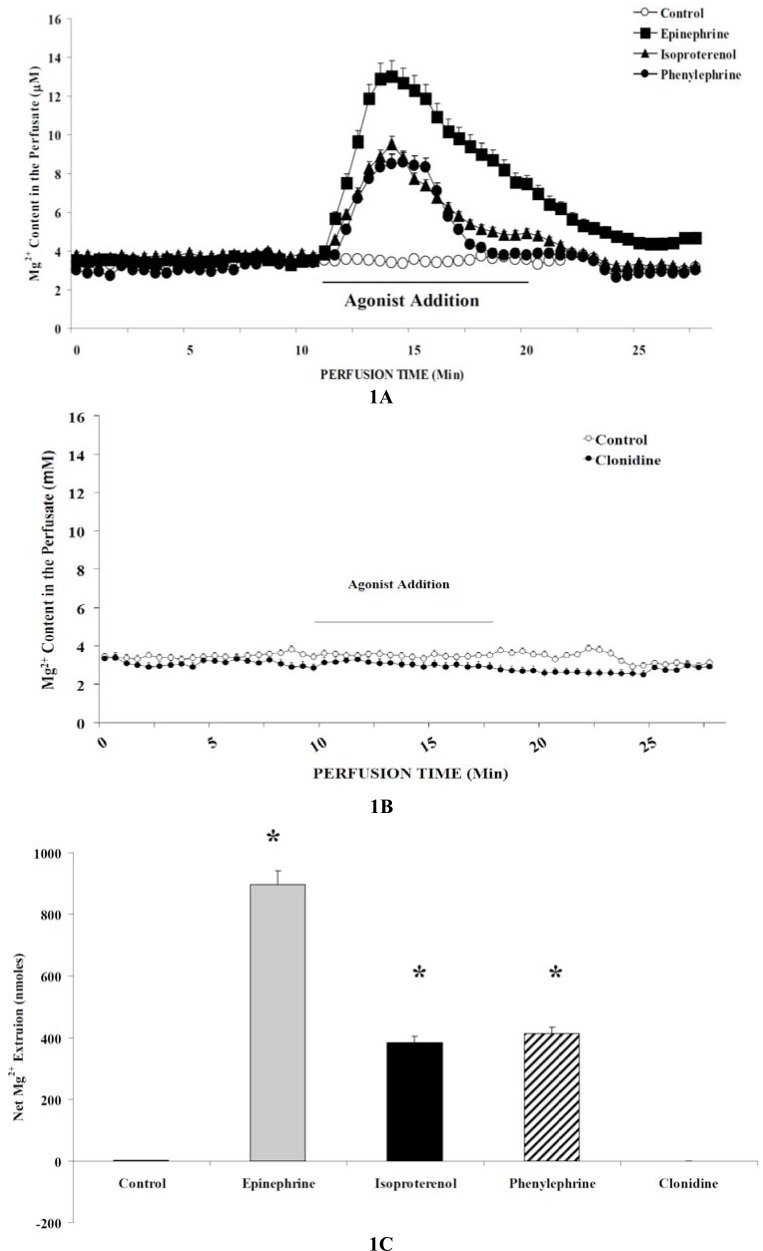
**Mg^2+^ extrusion from Langerdoff perfused hearts stimulated with various adrenergic agonist**. Rat hearts were perfused as reported under Materials and Methods. At the time indicated in the figures epinephrine (5 μM), phenylephrine (5 µM), isoproterenol (10 µM) or clonidine (5 µM) were added to the perfusion system for 10 min. Profile of hearts perfused with epinephrine, isoproterenol or phenyleprhine are reported in Fig. **1A**. Profile of hearts perfused with clonidine are reported in Fig. **1B**. Net Mg^2+^ extrusion under all conditions is reported in Fig. **1C**. Data are means ± SE of 6 hearts for each experimental condition. All the data points relative to Mg^2+^ extrusion following epinephrine, isoproterenol or phenyleprhine addition are statistically significant versus the corresponding control values. Labeling is omitted for simplicity.

Administration of the β-adrenoceptor agonist isoproterenol to perfused hearts resulted in a significant time and dose-dependent extrusion of Mg^2+^ into the perfusate (Fig. **1A**). A qualitatively similar extrusion was also induced by the α_1_-adrenoceptor agonist phenylephrine (Fig. **1A**) but not by the α_2_-adrenoceptor agonist clonidine (Fig. **1B**). Administration of the mix adrenergic agonist epinephrine (Fig. **1A**) resulted in a Mg^2+^ extrusion that was quantitatively equivalent to the amounts of Mg^2+^ mobilized by the combined stimulation of β- and α_1_-adrenergic receptors (Fig. **1C**). Further comparison indicated that the amount of Mg^2+^ extruded by β-adrenoceptor stimulation was quantitatively similar to the extrusion elicited by α_1_-adrenoceptor stimulation (Fig. **1C**). Taken together, these data suggest that cardiac myocytes possess at least two cellular Mg^2+^ pools that can be mobilized by the stimulation of α _1_- and β-adrenergic receptors, respectively. 

**Fig. (2):**
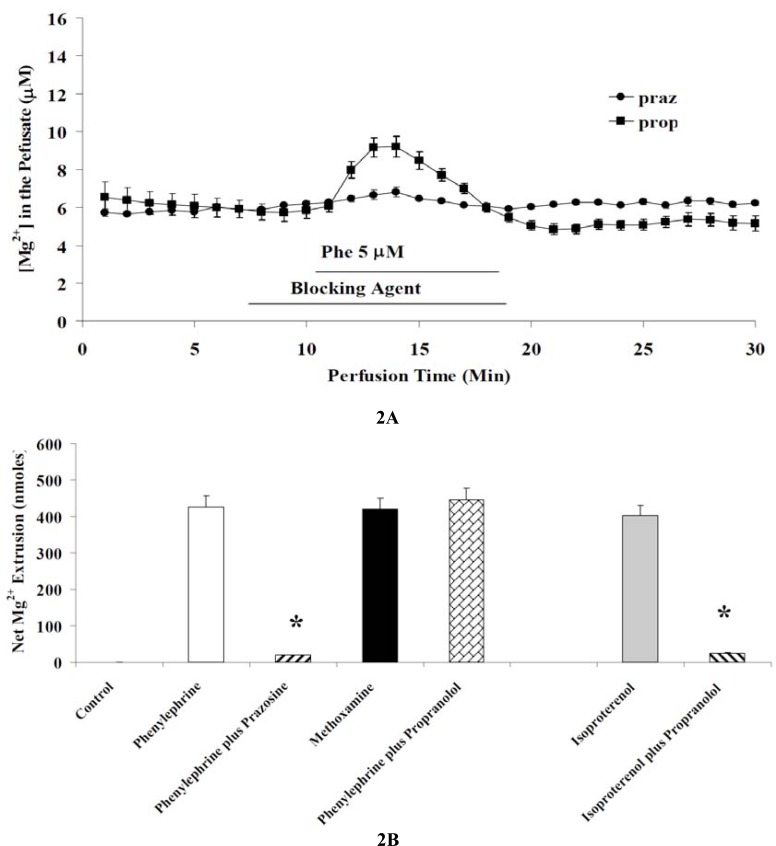
Mg^2+^ extrusion from rat hearts perfused with α_1_- and β-adrenergic receptor agonist in the presence of inhibitory agents. Rat hearts perfused as reported under Materials and Methods were stimulated by addition of phenylephrine (5 µM) or isoproterenol (10µM) in the presence of prazosin (1 µM) or propranolol (5 µM). Alternatively, hearts were perfused with methoxamine (5µM). Profile of hearts perfused with phenyleprhine in the presence of prazosin or propranolol are reported in Fig. **2A**. Net Mg^2+^ extrusion under all experimental conditions is reported in Fig. **2B**. Data are means ± SE of 6 hearts for each experimental condition. Fig. 2A: All the data pointμs relative to Mg2+ extrusion following phenyleprhine addition in the presence of prorpanolol are statistically significant versus the control values reported in Fig. 1A. Labeling is omitted for simplicity. Fig. **2B**: *Statistically significant as compared to the samples in the absence of inhibitor.

We have previously reported [8] that Mg^2+^ extrusion from cardiac myocytes is an active process resulting from β-adrenergic receptor engagement and not an epiphenomenon associated with cardiac contractility. In agreement with this observation, selective inhibition of α_1_- and β-adrenergic receptor by prazosin (Fig. **2A** and **2B**) and propranolol (Fig. **2B**) resulted in the inhibition of Mg^2+^ extrusion elicited by phenylephrine and isoproterenol, respectively. In contrast, pre-treatment of hearts with clonidine, which stimulates α_2_-adrenergic receptor and decreases cellular cAMP level, was ineffective at preventing isoproterenol-mediated Mg^2+^ extrusion (data not shown), in keeping with the limited representation of these receptors within the cardiac tissue [21] 

**Fig. (3):**
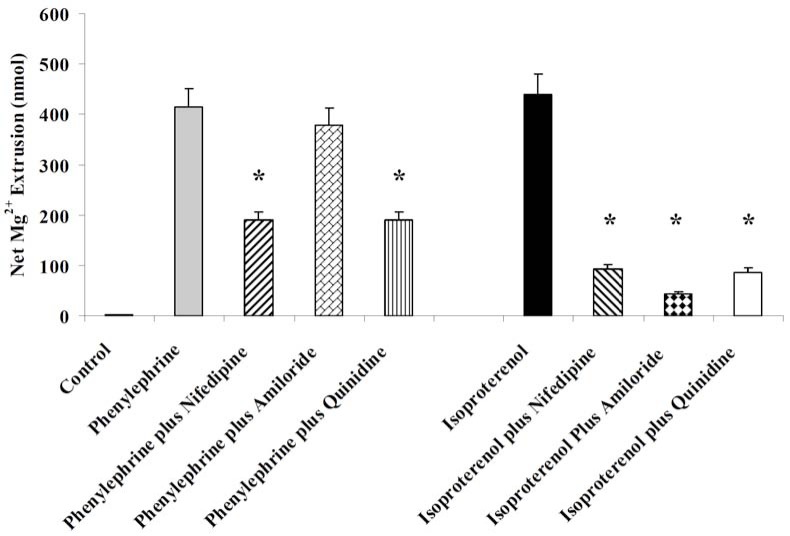
Mg^2+^ extrusion from rat hearts perfused with α^1^- and β-adrenergic receptor agonist in the presence of inhibitory agents. Rat hearts perfused as reported under Materials and Methods were stimulated by addition of phenylephrine (5 µM) or isoproterenol (10µM) in the presence of nifedipine (5 µM), amiloride (300 µM) or quinidine (250 µM). Net Mg^2+^ extrusion under all experimental conditions is reported. Data are means ± SE of 6 hearts for each experimental condition. *Statistically significant as compared to the Control sample.

Data in the literature indicate that β-adrenergic stimulation induces Mg^2+^ extrusion via cAMP-dependent activation (phosphorylation) of the Na^+^/Mg^2+^ exchanger [8,12,14], a process that can be inhibited by the administration of Rp-cAMP [22], or amiloride [16,23], which block adenylyl cyclase and the Na^+^/Mg^2+^ exchanger, respectively. To determine whether the Na^+^/Mg^2+^ exchanger was the ultimate mechanism responsible for Mg^2+^ extrusion following phenylephrine administration, phenylephrine stimulation was repeated in the presence of 300μM amiloride. The results, reported in (Fig. **3**), showed that α_1_-adrenoceptor mediated Mg^2+^ extrusion was not significantly inhibited by amiloride. Administration of quinidine, another non-specific Na^+^/Mg^2+^ exchanger inhibitor, however, resulted in a 40%-45% inhibition of Mg^2+^ extrusion (Fig. **3**).

Because α_1_-adrenergic stimulation in other organs (e.g. liver) requires extracellular Ca^2+^ [24], hearts were stimulated by phenylephrine in the presence of 1μM nifedipine. The results reported in (Fig, **3**), indicate that this dose of L-type Ca^2+^ channel inhibitor [25], while maintaining fairly normal beating hearts in the Langerdoff perfusion system, reduced Mg^2+^ extrusion by ~50%. Consistent with this observation, hearts perfused with phenylephrine in the presence of reduced [Ca^2+^]_o_ (i.e. 0.5mM) presented a reduced Mg^2+^ extrusion (187.56±6.82 nmol, n= 4, p<0.05) as compared to hearts perfused with physiological [Ca^2+^]_o_ (Fig. **1**). 

**Fig. (4):**
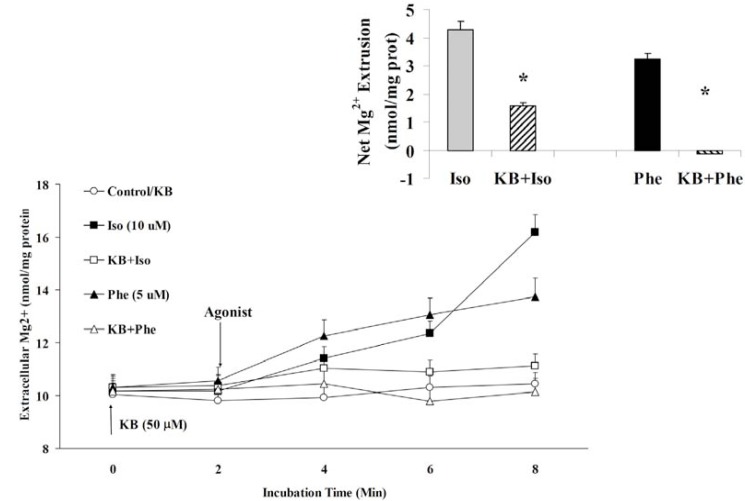
Mg^2+^ extrusion from collagenase dispersed cardiac ventricular myocytes incubated in the presence of α_1_- and β-adrenergic receptor agonist and KB-R7943. Cardiac ventricular myocytes were isolated by collagenase digestion and stimulated by addition of phenylephrine (5 µM) or isoproterenol (10 µM) as reported under Materials and Methods in the presence of KB-R7943 (50 µM). Net Mg^2+^ extrusion at time t = 8 (i.e. 6 min after agonist addition is reported for comparison in inset. Data are means ± SE of 9 preparations, each tested for all experimental conditions for the inset and 4 preparations for incubation profile. *Statistically significant as compared to the samples in the absence of inhibitor.

Results qualitatively to those reported in perfused hearts were observed in collagenase-dispersed cardiac myocytes incubated under similar experimental conditions (Table **1**). Table **1** and also (Fig. **4**) show that pretreatment of cardiac myocytes with KB-R7943, a fairly specific inhibitor of the Na^+^/Ca^2+^ exchanger in reverse mode [26], abolished to a significant extent the Mg^2+^ extrusion elicited by isoproterenol or phenylephine stimulation. 

**Fig. (5):**
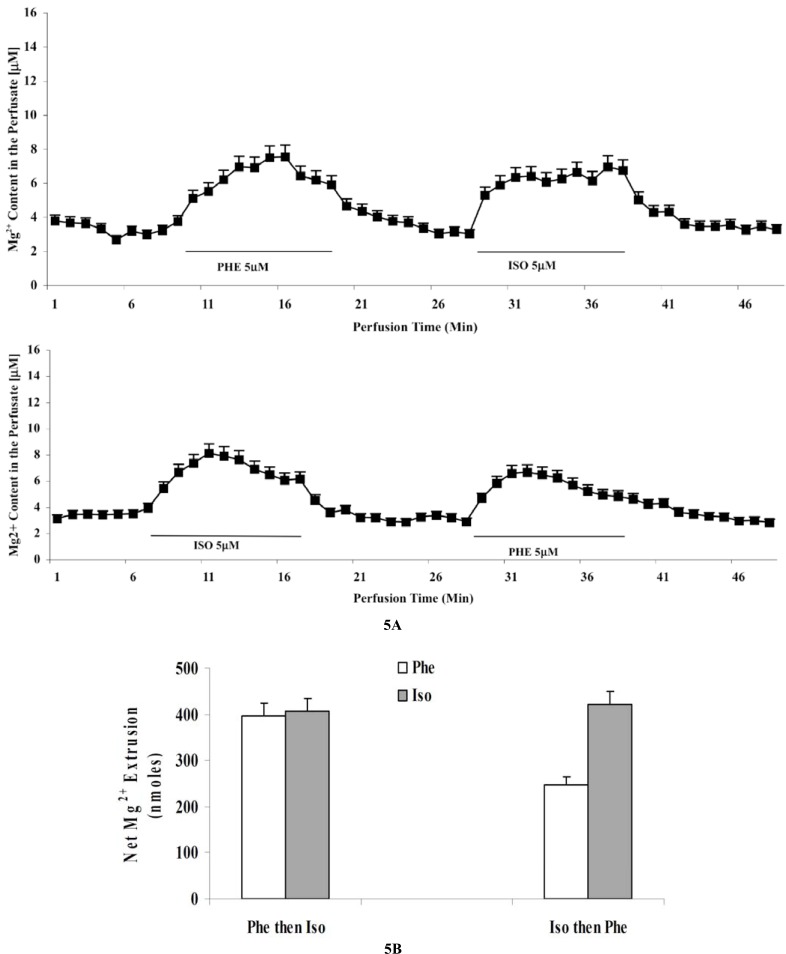
**Mg^2+^ extrusion from perfused hearts stimulated in sequence with α_1_- and β-adrenergic receptor agonist**. Rat hearts were perfused in sequence with phenylephrine (5 µM) and isoproterenol (10 µM) or with these agents in reverse order (Fig. **5A**). Net Mg^2+^ extrusion under these experimental conditions is reported in Fig. **5B**. Data are means ± SE of 6 hearts for each experimental condition.

The data reported in (Fig. **1**) suggest the presence of two distinct Mg^2+^ cellular pools targeted by α_1_-and β-adrenergic receptor, respectively. Thus, we assessed whether these two pools could be sequentially mobilized in the same heart by phenylphrine and isoproterenol administered in varying sequence order and with a varying time interval between stimulations. Fig. **5** shows the results of heart’s double perfusion with the two adrenergic agonists administered at 10 min interval from each other. To facilitate comparison, the net amount of Mg^2+^ extruded by each agonist is reported in (Fig. **5B**). As we can observe, administration of phenylephrine as the first agent does not affect the subsequent release of Mg^2+^ induced by isoproterenol. Administration of isoproterenol as the first agent, however, decreases the amplitude of phenylephrine-induced Mg^2+^ extrusion by approximately 40% (Fig. **5B**). Administration of epinephrine as the first agent, instead, decreases the amplitude of phenylephrine-induced Mg^2+^ extrusion by 88%, and that induced by isoproterenol by approximately 50% (Table **2**). Lastly, we varied the interval between administrations of β- and α_1_-adrenergic agonist. The results reported in (Table **2**) indicate that shortening the interval between the two stimulations resulted in a larger phenylephrine-induced Mg^2+^ extrusion whereas extending the interval resulted in a smaller Mg^2+^ extrusion. Administration of amiloride at the time of isoproterenol addition abolished the Mg^2+^ extrusion elicited by β-adrenergic receptor stimulation, and enlarged the subsequent extrusion elicited by phenylephrine (Table **2**). When the sequence of agonist was reverse, amiloride did not abolished the effect of phenylephrine, as already observed in (Fig. **3**), but markedly inhibited isoproterenol effect. Administration of quinidine, instead, resulted in a partial inhibition of both α_1_- and β-adrenergic receptor stimulations, as reported previously (Table **2**). 

**Fig. (6):**
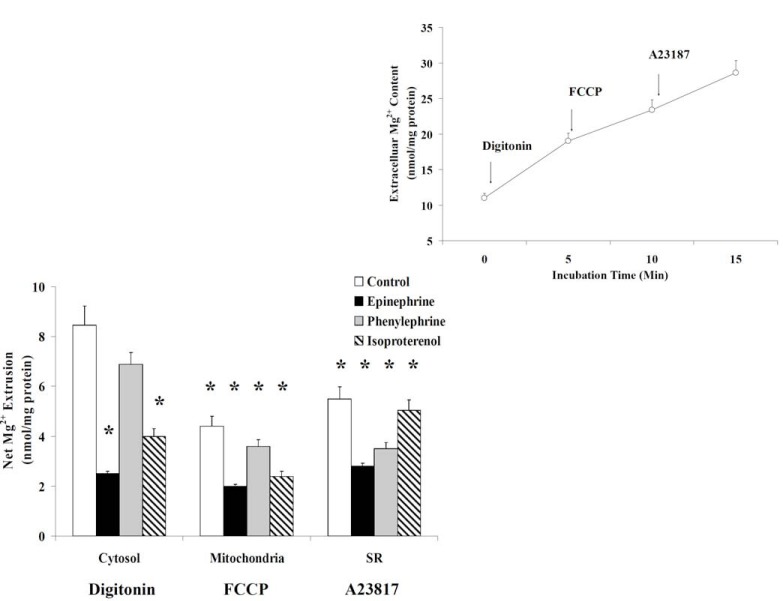
Mg^2+^ compartmentation in collagenase dispersed cardiac ventricular myocytes following stimulation by α_1_- and β-adrenergic receptor agonists. Cardiac ventricular myocytes were isolated by collagenase digestion and stimulated by addition of epinephrine (5 µM) phenylephrine (5 µM) or isoproterenol (10 µM) for 6 min. Myocytes were then sequentially treated with digitonin (50 µg/ml) FCCP (2 µg/ml) and ionophore A23187 (2 µg/ml) as reported under Materials and Methods, and as depicted in Figure inset. Net Mg^2+^ extrusion elicited by any of these agents is reported in the Figure. Data are means ± SE of 7 preparations, each tested for all experimental conditions. *Statistically significant as compared to the control values.

Because we lack advance techniques to detect in situ changes in cellular Mg^2+^ content and possible translocation from one cellular compartment to another, we resorted to use cardiac ventricular myocytes stimulated by adrenergic agonist *in vitro*, and sequentially treated with digitonin, mitochondrial uncoupler, and ionophore as indicated under Materials and Methods (Fig. **6**, inset) to mobilize Mg^2+^ from cytoplasm, mitochondria and non-mitochondria compartments. We have already used this approach in other cells with satisfactory results [27]. A typical incubation profile of cardiac myocytes treated with the three Mg^2+^-mobilizing agents is reported as an onset in (Fig. **6**). Fig. **6** also reports an estimate of the absolute content of Mg^2+^ mobilized by digitonin, uncoupler (FCCP) and A23187 ionophore from unstimulated cardiac myocytes and from cells previously treated for 8 min with phenylephrine, isoproterenol, and epinephrine. As the results indicate, phenylephrine stimulation largely mobilized Mg^2+^ from non-mitochondrial compartments (largely the sarcoplamisc reticulum) while isoproterenol stimulation depleted Mg^2+^ from cytoplasm and mitochondria. Consistent with its role of mix agonist, epinephrine mobilized Mg^2+^ from all compartments. Similar protocol was also applied to cardiac myocytes previously stimulated with phenylephrine or isoproterenol for 6 min. The results reported in (Fig. **6**) are suggestive of a Mg^2+^ redistribution among compartments based upon the type and duration of the stimulus applied.

## DISCUSSION

Elevated or persistent adrenergic stimulation of cardiac cells has been associated with increased incidence of cardiac arrhythmias. Adrenergic stimulation has been reported to induce Mg^2+^ extrusion from various tissues including cardiac myocytes [8-11]. Because most of these studies have utilized epinephrine or isoproterenol, they have focused on -adrenergic receptors [8-11]. β the stimulatory effect of these agents on cardiac αCatecholamine, however, can also stimulate _1_-adrenergic receptor, the effect depending on the receptor representation in the tissue, the catecholamine of choice (i.e. norepinephrine or epinephrine), and the experimental conditions. For example, studies carried out in our laboratory indicate that stimulation of _1_-adrenergic receptors induce Mg^2+^ extrusion in liver cells, where the process is associated with glucose output [18,24].

In the present study we attempted to address two distinct issues. The relative effect of _1_-adrenergic receptorβ- and stimulation on Mg^2+^ extrusion and cellular Mg^2+^ redistribution among compartment as well as the effect of the anti-arrhythmic drug quinidine, which has been reported to inhibit Mg^2+^ extrusion through the Na^+^/Mg^2+^ exchanger [14].

### Adrenergic Receptor Stimulation and Mg^2+^ Redistributionα

Stimulation of α_1_- and β- adrenergic receptors results in two well distinct Mg^2+^ extrusion processes when stimulatory agents for each receptor class are administered individually. The administration of catecholamine, which stimulate both classes of adrenergic receptors with different specificity, results in a Mg^2+^ extrusion equivalent to the sum of the amounts released by phenylephrine and isoproterenol individually. This is consistent with the notion that phenylephrine and isoproterenol target two distinct cellular Mg^2+^ pools within the cardiac myocytes. This observation is further congruent with the notion that β-adrenergic receptor stimulation mobilizes Mg^2+^ predominantly from cytoplasm and mitochondria whereas α_1_-adrenergic receptor stimulation mobilizes Mg^2+^ predominantly from the reticular pool (present data, and also [24, 27]. Stimulation of adrenergic receptors also results in cellular Mg^2+^ redistribution, which occurs concomitantly with the Mg^2+^ extrusion across the plasma membrane. Utilization of the mix agonist catecholamine prevents us from appreciating this redistribution, the process being more evident when specific adrenergic agonists are used in sequence. This is consistent with the observation that the sequential stimulation of the same class of adrenergic receptor with a short interval in between additions results in the progressive reduction in the amplitude of Mg^2+^ extrusion [8]. An increase in extracellular Mg^2+^ has been associated with a reduction in cardiac action potential amplitude and duration via inhibition of L-type Ca^2+^ channels and Na^+^ channels [9]. Less clear is the physiological significance of cellular Mg^2+^ redistribution following α_1_-adrenergic stimulation, mostly because of the novelty of our observation. Is this redistribution indicative of functional changes within individual compartments, such as regulation of mitochondrial dehydrogenases for energetic purposes [28], and reticular Ca^2+^ release from IP_3_ [29] and ryanodine receptor [30]? Or does it represent a compensatory mechanism to avoid major depletion of a cellular pool? Moreover, how is Mg^2+^ redistribution regulated and coordinated? Is the process regulated directly by the Mg^2+^ loss from a specific compartment? Or is there a role for post-receptor cellular signaling? For example, the increase in cytoplasmic cAMP level following β-adrenergic receptor stimulation appears to have a role in mediating Mg^2+^ mobilization from mitochondria [31], but it is unknown as to whether cAMP has any effect on the mitochondrial Mrs2 channel that allows Mg^2+^ entry into the organelle [32], thus favoring its subsequent redistribution. Similar questions can be posed for changes in reticular Mg^2+^ pool, for which limited information about Mg^2+^ transport and regulation is available. Our current technical inability to assess these events in real time prevents us from conclusively answering these questions in the context of this study. Further studies and experimental approaches need to be designed and implemented to this purpose. 

### Role of Anti-Arrhythmic Drug

An interesting observation generated from our studies is that the anti-arrhythmic drug quinidine inhibits by approximately 40-45% the Mg^2+^ extrusion elicited by α_1_- and β-adrenergic stimulation, consequently affecting the amplitude of the associated cellular Mg^2+^ redistribution. Quinidine, but also amiloride and imipramine have largely been used to inhibit Mg^2+^ extrusion in various experimental models although their mechanism of action is non-specific [14,23]. Our data, however, indicate that in the case of cardiac myocytes the effect of quinidine is more diffuse than that of amiloride, which we used for comparison throughout our study, in that quinidine affects the Mg^2+^ extrusion elicited by both α_1_- and β-adrenergic receptor to a comparable extent. In comparison, amiloride only inhibits the β-adrenenergic receptor mediated Mg^2+^ extrusion. This observation suggests that quinidine acts mainly by affecting membrane potential by inhibiting many Na^+^ transport mechanisms and also to a lesser extent K^+^ channels [17]. In turn, the changes in membrane potential can affect the operation of the Mg^2+^ extrusion mechanism. In this respect, it has to be kept in mind that although the Na^+^/Mg^2+^ exchanger constitutes the most abundant and perhaps most effective mechanism to extrude Mg^2+^ in cardiac cells following an increase in cellular cAMP level, it is not the only mechanism operating in the sarcolemma. Data obtained in isolated cardiac ventricular myocytes [16] and isolated sarcolemmal vesicles [12] indicate the operation of another exchanger with electroneutrally couples Mg^2+^ extrusion to Ca^2+^ (and other divalent cations). Interestingly, the operation of this extrusion mechanism is independent of cAMP-mediated phosphorylation, and can be related albeit indirectly to the activation of the α_1_-adrenergic receptor and associated Ca^2+^-mediated signaling [21,22], including Ca^2+^ entry across the cell membrane. This aspect will be consistent with the reduced phenylephrine-induced Mg^2+^ extrusion that occurs in cardiac cells in the presence of nifedipine, a L-type channel inhibitor commonly used under experimental and clinical conditions. Irrespective of the mechanism involved, it appears evident that quinidine and nifedipine, and perhaps other agents of similar classes, block Mg^2+^ extrusion and also cellular Mg^2+^ redistribution, thus affecting the level of Mg^2+^ retained within organelles and associated metabolic functions. In this contest, it would be important to assess in future studies to which extent prevention of Mg^2+^ extrusion and redistribution by anti-arrhythmic drugs and Ca^2+^ channel blockers is integral part of the pharmacological effectiveness of these therapeutic agents. 

## CURRENT & FUTURE DEVELOPMENTS

Our results indicate that catecholamine administration induces a major efflux of Mg^2+^ from cardiac ventricular myocytes, which results in dynamic changes in total and compartmentalized Mg^2+^ pools within the myocytes. In addition, our data suggest that prevention of Mg^2+^ extrusion and redistribution may represent an integral component of the effectiveness of at least some anti-arrhythmic drugs and Ca^2+^-channel blocking agents. Additional studies both at the experimental and the clinical levels are necessary to confirm our initial observation and its pharmacological relevance. If confirmed, this observation might result in patenting (some of) the existing anty-arrhythmic and L-type-Ca^2+^ channel blockers as Mg^2+^ preserving agents. Additionally, it might lead to the development of new therapeutic agents that are more effective at preventing cellular Mg^2+^ loss, with consequent enhanced effectiveness and reduced toxicity or intrinsic arrhythmogenicity, which often limit their pharmacologic use. 

## Figures and Tables

**Table 1:** Mg^2+^ Extrusion from Collagenase Dispersed Cardiac Ventricular Myocytes Stimulated by Adrenergic agonists in the Presence of Various Inhibitory Agents.

	Before agonist Addition	6 Min after Agonist Addition
Control	10.16±0.35	10.14±0.29
Epinephrine (Epi)	9.98±0.24	16.05±0.56*
Phenylephrine (Phe)	10.08±0.27	13.75±0.48*
Isoproterenol (Iso)	10.10±0.32	14.98±0.42*
		
Epi ± Amiloride		12.21±0.16#
Epi ± Quinidine	12.02±0.24#
Epi ± Nifedipine		13.21±0.41#
Epi ± Prazosin		13.35±0.55#
Epi ± Propranolol		12.40±0.19#
		
Phe ± Amiloride		13.57±0.65
Phe ± Quinidine		11.06±0.36#
Phe ± Nifedipine		11.24±0.51#
Phe ± Prazosin		10.89±0.23#
Phe ± Propranolol		13.68±0.44
		
Iso ± Amiloride		11.15±0.33#
Iso ± Quinidine		11.82±0.28#
Iso ± Nifedipine		11.67±0.40#
Iso ± Prazosin		14.76±0.11
Iso ± Propranolol		10.89±0.30#

Results are reported as nmol/mg protein. Data are means ± S.E. of 6 preparations tested for each experimental condition.

* Statistically significant vs. Control (p<0.05).

# Statistically significant vs. corresponding value without inhibitor (p<0.05).

**Table 2:** Mg^2±^ Extrusion from Perfused Hearts Sequentially Stimulated by Adrenergic agonists at Various Intervals

	1st	2^nd^ Addition after			
		5 min	10 min	15 min	20 min
Phe	396±15				
Iso following Phe		411±21	406±12	391±29	378±35
Iso	420±31				
Phe following Iso		347±34	248±43#	200±21#	158±51#

Results are reported as net Mg^2+^ extrusion (nmoles per area under the curve). Data are means ± S.E. of 6 preparations for each experimental condition.

# Statistically significant vs. corresponding value administered as the first dose (p<0.05).
